# Investigating the transfer of physical stimuli to the dreamed body: A comparative study of muscular, vestibular, and haptic stimulation on dream incorporation

**DOI:** 10.1371/journal.pone.0358438

**Published:** 2026-10-14

**Authors:** Emma Peters, Jakob Poehlmann, Xinlin Wang, Kathrin Fischer, Stefano Maraschin, Gian Jäger, Gabriel Bolliger, Ralf Kredel, Simon Maurer, Martin Dresler, Daniel Erlacher

**Affiliations:** 1 Institute of Sports Science (ISPW), University of Bern, Bern, Switzerland; 2 School of Health Professions, Institute of Physiotherapy, Zurich University of Applied Sciences (ZHAW), Winterthur, Switzerland; 3 Department of General Psychology, University of Padova, Padua, Italy; 4 Department of Developmental Psychology and Socialisation, University of Padova, Padua, Italy,; 5 Technology Platform for Research, Faculty of Human Sciences, University of Bern, Bern, Switzerland; 6 Donders Institute for Brain, Cognition and Behaviour, Radboud University Medical Centre, Nijmegen, The Netherlands; Institut VEDECOM, FRANCE

## Abstract

The relationship between the dreamed body and the physical body remains an open question in sleep and dream research. This study examined whether somatosensory stimulation applied during REM sleep modulates the corresponding dream content under controlled conditions. Using a within-subject design, we compared three stimulation modalities—electrical muscular, galvanic vestibular, and haptic vibration—each paired with a modality-specific sham stimulation. Twenty-four participants completed one adaptation night followed by three consecutive experimental nights in the sleep laboratory. Dream reports were collected following rapid eye movement sleep awakenings after stimulation or sham periods, yielding a total of 120 reports. Dream incorporation of bodily sensations and actions was observed across all conditions; however, generalized linear mixed-effects analyses revealed no statistically significant differences between stimulation and sham conditions for any modality. No evidence for reliable modality-specific dream incorporation was observed under rigorous sham-controlled conditions. Rather than demonstrating robust stimulation-specific effects, these findings suggest that contextual and expectancy-related influences substantially contribute to bodily dream content under laboratory conditions while highlighting methodological challenges in dream incorporation research. By combining multiple somatosensory stimulation modalities with rigorous sham-controlled procedures, the present study provides a methodological benchmark for future investigations of dream incorporation and dream engineering.

## 1. Introduction

Experimental alteration of dream content using external stimulation during sleep has been explored for many decades, demonstrating that dream experiences are shaped not only by internal processes but also by sensory input from the environment and the body. External stimuli such as sounds, scents, light, temperature, or bodily sensations can infiltrate ongoing dream experiences and influence dream content, a phenomenon referred to as dream incorporation [1,2]. Investigating dream incorporation is central to understanding fundamental mechanisms of dream formation and the role of the physical body during sleep. Beyond basic research, controlled manipulation of dream content has been proposed as a foundation for dream engineering approaches, including clinical applications and interventions targeting dream experiences [3]. In addition, external stimulation during sleep has been used to induce lucid dreaming, a state in which the dreamer becomes aware of dreaming while remaining asleep [4]. Lucid dreaming has been discussed in relation to applications such as nightmare therapy, creative problem-solving, skill development, and personal exploration. [5–7].

### 1.1 The role of the sensorimotor cortex and bodily synchrony in dream experiences

Neurophysiological evidence indicates that the dreamed body is closely linked to the physical body. Dreamed actions, such as hand movements, have been shown to activate sensorimotor regions of the brain in patterns comparable to those observed during wakeful movement execution [8]. Building on this finding, non-invasive brain stimulation studies have demonstrated that modulating activity in the sensorimotor cortex can alter movement-related dream content. For example, transcranial direct current stimulation (tDCS) has been shown to influence movement-related cognition. While unilateral motor cortex stimulation increased motor imagery during wakefulness [9] and during rapid eye movement (REM) sleep [10], bihemispheric stimulation over the primary motor cortex reduced reports of dream movements without affecting tactile or vestibular sensations [11]. These findings suggest that sensorimotor cortical activity contributes directly to the generation and modulation of bodily experiences in dreams, supporting the idea that bodily signals can influence dream content.

In addition to neural activations, physiological synchrony between dreamed experiences and the physical body has been repeatedly observed. Dreamed eye movements and gaze direction are reflected in REM eye movements [12,13], and emotional facial expressions occurring in dreams are mirrored by facial muscle activity during REM sleep [14]. More broadly, autonomic and muscular responses, such as changes in heart rate, respiration, and muscle tone, often correspond to the emotional and motor content of dreams [15]. Together, these findings suggest a bidirectional interaction between the dreamed body and the physical body, in which bodily signals can shape dream experiences and dreamed actions can elicit measurable physiological responses. This bidirectional coupling provides a mechanistic basis for using external bodily stimulation as an experimental approach to investigate the incorporation of dream content.

### 1.2 Influence of somatosensory stimulation on dream content incorporation

A wide range of studies have examined the incorporation of external stimuli into dreams, including visual, auditory, olfactory, and somatosensory stimulation. [1,16]. Within this literature, somatosensory stimulation is of particular interest because it directly stimulates tactile, proprioceptive, muscular, or vestibular sensory systems through physical contact with the body, rather than indirectly through environmental sensory cues such as sounds or odors. Given the demonstrated coupling between bodily states and dream experiences, somatosensory stimulation represents a promising experimental approach for investigating how physical signals are translated into dream content. However, reported incorporation rates across studies vary widely, ranging from 0% to 90% [1,2], depending on stimulation type, timing, and methodology.

Early work demonstrated that electrical muscle stimulation applied during REM sleep can influence dream content. Koulack [17] reported increased bodily centrality and activity in dreams following somatosensory stimulation, particularly when stimulation occurred early in REM sleep. Tactile somatosensory stimulation has also been investigated using vibrotactile stimulation applied to the fingers, wrist, or ankle, with reported incorporation rates ranging from approximately 40–50% in dream reports [18]. However, these studies were based on small samples and, in some cases, lacked sham control conditions, making it difficult to disentangle true stimulus-specific incorporation from contextual or expectancy effects.

Vestibular stimulation has also been investigated as a means of influencing dream content. Leslie and Ogilvie [19] reported greater incorporation of vestibular sensations into dreams during rocking stimulation than under stationary control conditions, and this incorporation was associated with increased dream bizarreness. In contrast, studies that used upper-body tilting during naps did not observe clear differences in vestibular or somatosensory dream content between the tilted and horizontal conditions [20]. Vestibular sensations such as movement, balance disturbances, flying, or falling are frequently reported features of dream experiences, suggesting that the vestibular system may play a role in the formation of dream content [21]. Galvanic vestibular stimulation (GVS) provides a non-mechanical method to directly modulate vestibular afferents by applying electrical current over the mastoid bones [16] and reliably induces vestibular sensations during wakefulness. Despite its extensive use in fundamental vestibular research, GVS has not yet been applied to systematically examine how vestibular signals are incorporated into dream content during sleep.

### 1.3 Research question and hypotheses

Although previous research demonstrates that somatosensory stimulation can be incorporated into dream content, existing findings are difficult to compare due to small sample sizes, heterogeneous methodologies, and the frequent absence of sham control conditions. As a result, it remains unclear whether different forms of somatosensory stimulation selectively influence corresponding dream content, or whether reported incorporations primarily reflect non-specific effects of the experimental context. Moreover, few studies have directly compared multiple somatosensory modalities within the same individuals. The present study addresses this gap by systematically comparing three somatosensory stimulation modalities: electrical muscle stimulation (EMS), haptic vibratory stimulation (HS), and galvanic vestibular stimulation (GVS), using a within-subject design with a dedicated sham condition for each modality. By applying stimulation during REM sleep and collecting dream reports following awakenings, we aimed to assess whether stimulation would be incorporated into dream content and, if so, whether the incorporation was modality-specific. Based on the targeted sensory pathways of each stimulation method, we expected GVS to be associated with balance-related dream content (e.g., flying), EMS with bodily movement (e.g., limb movement), and HS with tactile or somatosensory experiences (e.g., pressure).

## 2. Materials and methods

### 2.1 Participants

Participants (n = 24; 12 female) were recruited through advertisements around the campus of Bern University and via the researchers’ social media groups and circles in Bern, Switzerland, from 01.11.2022 to 01.04.2023. Participants were healthy, aged 20–37 years (M = 26.5, SD = 4.97), and able to speak English, German, or Swiss-German. When applying for the study, participants were pre-screened online. They were excluded if they had a low dream recall rate (<3 per week), a late bedtime (>1 am), a long sleep latency (>1 hour), and finally, if they, or any family members, were diagnosed with epilepsy, sleep-related disorders, or current psychiatric disorders. The average dream recall was 4 days per week. An a priori sample size estimation was conducted to obtain an approximate indication of the number of participants required. Because no previous studies had applied comparable sham-controlled within-subject designs analyzed using generalized linear mixed-effects models, formal power estimation for the planned analyses was not feasible. Instead, we used published dream incorporation rates reported by Schredl [22], ranging from approximately 25% to 47%, to estimate a medium-to-large effect size (w = 0.35) using χ² assumptions. This calculation yielded a target of 79 observations. We acknowledge that this calculation served only as a rough estimate because observations within participants are not statistically independent and the final analyses were conducted using generalized linear mixed-effects models.

The study was performed in the sleep laboratory at the Institute of Sport Sciences (University of Bern). Participants were financially compensated. The Faculty of Human Sciences of the University of Bern approved the study ethically. Before the study, participants were informed of the study’s aim and signed a written consent form. Data protection was ensured by using a pseudonymous code for each participant.

### 2.2 Experimental setup

Each participant spent four consecutive nights in the sleep laboratory. The participant arrived at 9 pm each night and got ready for bed. Polysomnography (PSG) was primarily used for online identification of sleep stages and REM awakenings. Offline sleep staging was subsequently performed to obtain descriptive sleep parameters, but PSG signals were not analyzed for neurophysiological outcomes. The PSG setup used 16 disposable silver cup electrodes with the gUSBamp EEG system and the University of Bern’s custom experiment management system, Streamix. The sampling rate was 256 Hz. The PSG consisted of electroencephalography (EEG), electrooculography (EOG), and electromyography (EMG). PSG setup was applied according to the 10–20 system [22]. F3, F4, C3, C4, O1, O2, horizontal EOG1 and EOG2, chin EMG left (EMGL), and chin EMG right (EMGR), a reference (earlobe) and ground (Cz) electrode were used. The first night was an adaptation night aimed at helping participants get used to the environment and the PSG setup. During this first night, no stimulation devices were applied, and no awakenings were performed. In each of the following three nights, participants received one of the three stimuli (haptic, vestibular, muscle) during REM sleep periods, including sham stimulation trials. One stimulation device was applied per night, and the order of the stimulation methods and sham stimulation was randomized and counterbalanced, as shown in Fig 1.

**Fig 1:**
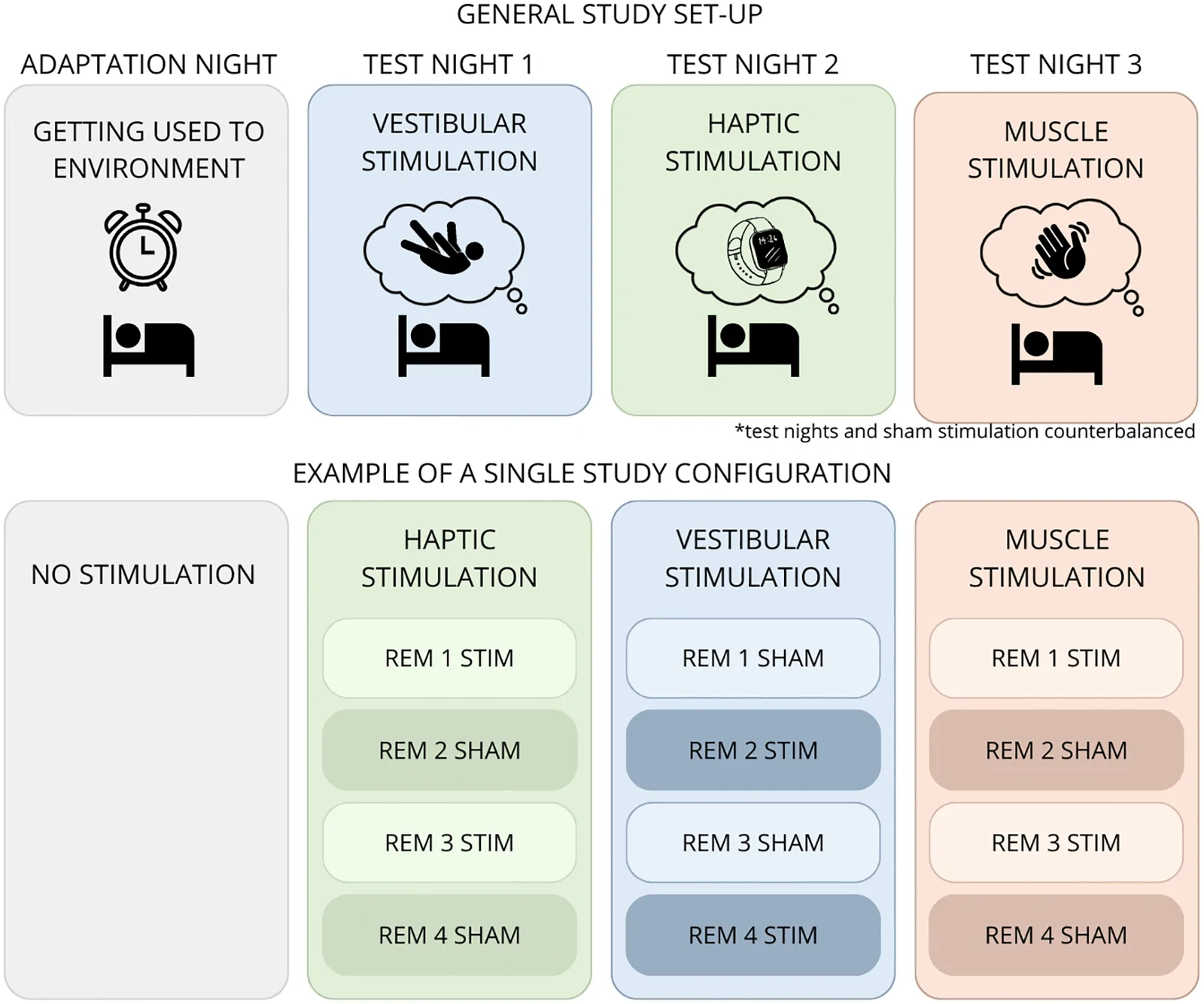
Top: the study overview with the adaptation night and the three stimulation nights. Bottom: an example of the configuration for a single participant with stim and sham order in REM phases balanced across the nights. In this specific configuration, the night order is haptic, vestibular, and muscle stimulation.

### 2.3 Stimulation methods

The three stimuli used were Galvanic Vestibular Stimulation (GVS), Electrical Muscle Stimulation (EMS), and Haptic Stimulation (HS). Each stimulation lasted for two seconds.

*(Bipolar binaural sinusoidal) Galvanic Vestibular Stimulation* was set up using the NeuroConn DC-Stimulator Plus (NeuroConn DC-STIMULATOR PLUS | Neurocare Technology, 2023) [23] and by attaching two 4.5x3.5mm carbon rubber electrode pads behind the ear over the mastoid bone. The electrodes were cut to size to enable precise application on the mastoid bone. Electrical stimulation over the mastoid bone stimulates the vestibular afferents [24]. This can induce a variety of vestibular sensations, depending on the position of the body and head in space, as well as the electrode placement [25]. To ensure a consistent stimulation experience across participants, we investigated GVS in detail. The effect of the stimulation on horizontal body position was tested, including positions on the back and on the side, with or without a pillow. In addition to spatial positioning, different stimulation parameters were tested, including stimulation intensity (μA), duration (s), frequency (Hz), waveform type (tDCS, tACS, sine, half-sine), and fade-in and fade-out times (s). We noticed that the stimulation frequency had the most consistent effect on perception: lower frequencies (0.5 Hz) resulted in a boat- or wave-like feeling, while higher frequencies (1–2 Hz) were perceived as a more abrupt, unnatural, and externally produced movement. The experiment was finally run with sinusoidal bi-polar stimulation (tACS). The stimulus parameters were set to a level where the participants felt a small sway sensation without feeling an uncomfortable skin sensation from the current behind the ear while sitting on the bed with their eyes open (intensity: varying from 1mA to 3mA peak-to-peak, frequency: 1 Hz, duration: 2s, phase:0*, fade in/out: 0s, number of cycles: 2*2pi). These parameters resulted in a quick left-right-left-right head sway.

*Electrical Muscle Stimulation* induced a movement of the wrist and fingers consisting of a flexion of the ring and pinky finger. To achieve this, two 50x100mm self-adhesive TENS-EMS gel electrodes were placed on the **volar side of the left forearm** targeting the flexor muscles responsible for ring-finger flexion. The **distal electrode** was positioned approximately **2–3 cm proximal to the wrist crease**, aligned longitudinally with the forearm on the ulnar side. The **proximal electrode** was placed **5–6 cm above** the distal electrode along the same axis. This placement corresponded to the anatomical course of the wrist flexor muscle group involved in flexion of the ring finger. For each participant, the electrode positions were slightly adjusted (within 1–2 cm) during wakefulness to achieve **clear flexion of the ring finger into the palm** at the lowest comfortable stimulation intensity. Once the finger movement was reliably produced, the electrodes were secured to prevent displacement during sleep. The device used to apply the electrical stimulation was the Rehamove 3 “ScienceMode” Device. The Rehamove 3 is a small handheld device that can be connected to a computer, enabling external stimulation triggering [26,27]. EMS was delivered with a pulse period of 20ms (corresponding to 50 Hz), no amplitude ramp (ramp = 0), and a waveform defined by a single stimulation point of 3 mA with a pulse duration of 500 µs; the stimulation was applied as a continuous pulse train lasting approximately 2 seconds.

*Haptic Stimulation* was applied using a home-made vibrotactile device. This consisted of a small plastic housing (approximately 6 cm in length with a diameter of 1.5 cm) with an eccentric rotating mass (ERM) vibration motor, operating at 3V with a frequency of 100–150 Hz. This was connected to a current-producing Lab Jack U3 LV that could be triggered from the control room to produce a slight vibration of the plastic tube. The vibrating device was attached to the left dorsal wrist, approximately 2–4 cm proximal to the wrist crease, with its long axis aligned parallel to the forearm using medical adhesive tape. The stimulation had a single fixed intensity. Before lights out, participants briefly experienced the vibration while awake to familiarize themselves with the sensation and confirm that it was clearly perceptible yet comfortable.

### 2.4 Stimulation protocol

REM periods were identified from the PSG recordings using the AASM criteria. REM onset was marked when low-amplitude mixed-frequency EEG activity, rapid eye movements in the EOG channels, and reduced chin EMG tone were present for at least one 30-second epoch. Only clearly identifiable and stable REM periods were used for stimulation. The stimulation was delivered during REM sleep using one of the randomly selected stimulation methods in a counterbalanced order. The first REM phase was disregarded, and REM awakenings were obtained from the second to the fourth REM periods, provided these periods could be clearly identified. For each of these REM periods, the experimenter waited 5, 10, or 15 minutes after verified REM onset to ensure a stable dream episode before starting the protocol. For each participant, we aimed to deliver both stim trials and sham trials per night, assigned according to a predefined counterbalancing schedule that distributed stim and sham conditions across the second, third, and fourth REM periods at the group level. However, the actual number of REM periods per night varied substantially across participants, and some participants did not enter all intended REM periods. When a planned REM period did not occur or was interrupted by wakefulness, the corresponding stim or sham trial was reassigned to the next available REM period rather than omitted. As a result of this variability, not all participants received the same number of trials, and the mapping between condition and REM period was not identical across individuals. Despite these individual-level differences, the predefined counterbalancing ensured that, at the group level, stim and sham trials were distributed across REM periods with comparable frequency.

Stimulation delivery was monitored in real time via the experiment management system Streamix (University of Bern), which logs device status and error messages throughout the recording. In one rare case, stimulation failed due to a loss of connection to the EMS device, as indicated by a system-level “device disconnected” error in the log files. The trial, however, was excluded after post-hoc polysomnography scoring revealed that it did not occur during REM sleep.

Every stim condition contained a maximum of 10 two-second stimulations, with a 30-second wait period between stimulations and after the final stimulation, resulting in a total intended duration of 320 seconds for a complete trial. The sham condition consisted of a timer that was set to match the duration of the entire stim condition. If the participant entered non-rapid-eye-movement (NREM) sleep, the protocol was aborted and restarted at the next REM onset. If the participant woke up completely due to arousal following stimulation or during the sham timer, the protocol was aborted, and the dream report was collected. When the participant remained in REM sleep throughout the 10 stimulations, the researcher awakened the participant via intercom and collected a dream report. Answers were recorded on audiotape and later transcribed. After collecting the dream report, the participants were allowed to go back to sleep; the researcher waited for the next REM phase and repeated the protocol.

### 2.5 REM awakenings

The REM awakenings were initiated by calling the participant by name, followed by a question about whether they were awake. When they reported being awake, the question: ‘What went through your head before you woke up?’ was asked. After the participant finished their report, the question: ‘Did you notice anything else?’ was asked to collect more detail.

### 2.6 Dream data analysis

After transcribing the audio dream reports into written form, they were processed according to a dream report cleaning Manual [28]. These include removing any words or sentences that do not directly refer to the dream content. Because the dream reports were collected in multiple languages, they were all translated into English using DeepL Translator to ensure high-quality, contextually appropriate translations. Finally, the dream reports were randomized and rated by two external, blind raters. To evaluate the dream reports, the raters used a Dream Content Analysis (DCA) Manual that included stimuli related to movement, balance, and somatosensory sensations. Each dream report was evaluated quantitatively across these categories. Any dreams reporting the participant being awake during stimulation or waking up as a result of stimulation were excluded.

For each of the three stimulation methods, one scale was selected to represent direct incorporation for that method. For GVS, the scale ‘General Balance-Related Activities’ was selected. This scale is phrased in the Dream Content Analysis Manual as: ‘Does the dreamer perform activities involving balancing and balance, e.g., flying, going over a narrow bridge? Does the dreamer have problems with balance, e.g., the occurrence of dizziness, fear of looking deep, etc?’. The scale thus combines all these parameters. For EMS: ‘Movement of the Arms’ was selected and was phrased as: ‘Does the dreamer perform activities that have to do with the arms, e.g., B., waving, manual labor, boxing? The activity should be explicitly named.’ Finally, for HS, the first scale: ‘Does the dreamer experience activities in which a tactile or somatosensory sensation/stimulus plays a role?’ was selected together with a second scale: ‘Mark the parts of the body where the sensation occurred in the first scale.’ If the first scale was scored as a 1 in combination with the mention of the arms in the second scale, then the final score for ‘Tactile or Somatosensory Sensations in the Arms’ was scored as 1. The dream incorporation rate was calculated by summing all scores for each stimulation category and correcting for the number of dreams per category.

### 2.7 Polysomnography data

Polysomnography (PSG) data were scored using U-Sleep, a validated deep-learning–based automated sleep staging algorithm trained on large, manually scored datasets. PSG recordings (EEG, EOG, and EMG channels) were exported in standard format and processed offline using U-Sleep, which assigns sleep stages (Wake, N1, N2, N3, and REM) in 30-s epochs. Automated scoring was subsequently used to obtain the sleep parameters shown in Tables 1 and 2.

**Table 1:** The PSG data on the adaptation nights and test nights for all 24 participants.

	Nights											
	Adaptation			1. Night			2. Night			3. Night		
Total bed time (min)	484.8	±	134.6	497.3	±	52.3	502.0	±	72.2	515.2	±	67.6
Total sleep time (min)	392.7	±	121.2	392.9	±	52.3	400.6	±	80.8	417.2	±	67.6
Sleep efficiency (%)	82%	±	12%	78%	±	18%	80%	±	14%	81%	±	11%
REM total time (min)	41.1	±	28.1	52.1	±	29.6	53.5	±	25.7	49.0	±	30.3
REM% SPT	8%	±	5%	10%	±	5%	11%	±	5%	10%	±	6%
Wake% SPT	18%	±	12%	22%	±	18%	20%	±	14%	19%	±	11%
Stage 1% SPT	3%	±	3%	4%	±	2%	3%	±	2%	3%	±	1%
Stage 2% SPT	52%	±	14%	47%	±	12%	47%	±	8%	47%	±	12%
Stage 3% SPT	19%	±	9%	16%	±	7%	19%	±	7%	18%	±	9%

REM: Rapid Eye Movement sleep, SPT: Sleep Period Time

**Table 2:** The PSG data on the three conditions for all 24 participants.

	Conditions								
**EMS**			**GVS**			**HS**		
Total bed time (min)	507.9	±	54.5	502.9	±	37.8	497.1	±	82.1
Total sleep time (min)	398.0	±	92.6	412.0	±	61.6	390.4	±	85.3
Sleep efficiency (%)	78%	±	18%	82%	±	10%	79%	±	14%
REM total time (min)	50.1	±	25.4	53.6	±	26.9	53.3	±	30.0
REM% SPT	10%	±	5%	11%	±	5%	11%	±	6%
Wake% SPT	22%	±	18%	18%	±	10%	21%	±	14%
Stage 1% SPT	4%	±	2%	4%	±	2%	3%	±	1%
Stage 2% SPT	47%	±	12%	49%	±	5%	47%	±	8%
Stage 3% SPT	18%	±	7%	18%	±	7%	18%	±	8%

EMS: Electrical Muscle Stimulation, GVS: Galvanic Vestibular Stimulation, HS: Haptic Stimulation, REM: Rapid Eye Movement sleep, SPT: Sleep Period Time.

For the final data set, dream reports were excluded if wakefulness was detected during the stimulation trials or if stimulation happened during NREM sleep. An average of 9.11, 10.0, and 8.67 stimulations were presented in the EMS, HS, and GVS stimulation trials, respectively.

### 2.8 Statistics

Dream incorporation outcomes were binary and repeatedly measured within participants. Data were therefore analyzed using generalized linear mixed-effects models (GLMMs) with a binomial distribution and logit link function (GAMLj module, jamovi). For arm movement and tactile-arm incorporation, analyses were restricted to EMS and HS conditions across the two stimulation conditions (STIM vs SHAM). Balance-related incorporation was analyzed separately for GVS trials only, with stimulation condition as a fixed effect and participant as a random intercept. Simple-effects contrasts comparing stimulation and sham conditions within each modality were derived from the fitted models. Statistical significance was assessed using χ² tests, and no post hoc power analyses or effect size interpretations were performed for non-significant results.

## 3. Results

### 3.1 Awakenings and dream reports

In 72 experimental nights, excluding adaptation nights, a total of 184 awakenings were performed in REM sleep, 96 in the stim condition, and 88 in the sham condition. 65, 61, and 58 awakenings were performed in the EMS, GVS, and HS conditions, respectively. The discrepancy between the planned number of 288 awakenings and the final number of awakenings was due to a lack of usable REM periods. A total of 165 dream reports were recorded, which is equivalent to a dream recall rate of 89.7%. Forty-five dream reports were excluded due to awakening during stimulation, as indicated by the dream reports and the PSG data; the remaining 120 dream reports were included in the analysis. 45 Dreams were included in the stim condition and 75 in the sham condition. One participant did not report any dreams throughout the experiment. The average dream length of the EMS stim group was 59.2 words; for EMS sham, this was 68.7. For GVS STIM and GVS sham, this was 91.9 and 88.1 words, respectively. Finally, HS stim and HS sham had an average dream length of 72.7 and 79.7 words, respectively.

### 3.2 Dream incorporation

Using the DCA manual, a variety of scales were investigated. The DCA manual and the results are in the Appendix. For our research question, we will focus on three main scales that represent the relevant stimulation method. For EMS and HS, these were ‘Movement of the Arms’ and ‘Tactile or Somatosensory Sensations in the Arm’, and for GVS, this was ‘General Balance-Related Activities’. EMS and HS are tested on the same scales as they have overlapping stimulation experiences, whereas GVS stimulation elicited a completely independent experience. The average consensus of the two raters for the selected scales was 88.96%. The final consensus was reached through discussion of the remaining open items. Fig 2 presents the dream incorporation rate (DIR), defined as total dream incorporation, corrected for the number of dreams collected per condition.

**Fig 2:**
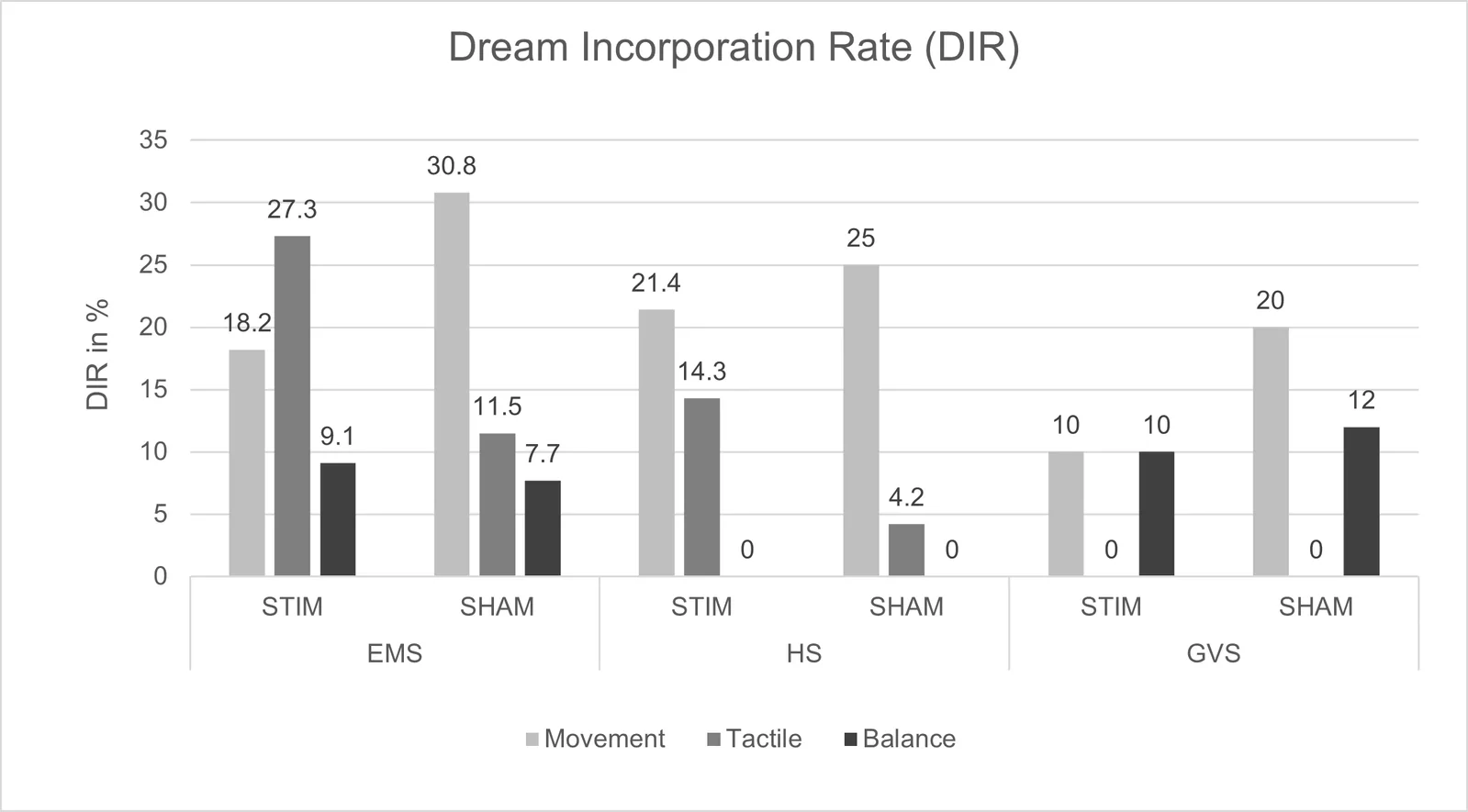
The Dream Incorporation Rate (DIR) of ‘Movement of the Arms,’ Tactile or Somatosensory Sensations in the Arms,’ and ‘General Balance-Related Activities’ per stimulation condition as a percentage of dreams in the condition. ST = stimulation condition, SH = sham condition. EMS: Electrical Muscle Stimulation, GVS: Galvanic Vestibular Stimulation, HS: Haptic Stimulation, STIM = stimulation condition, SHAM: sham stimulation condition.

For EMS, arm movement incorporation was observed in 18.2% of dreams in the stimulation condition and 30.8% in the sham condition. Tactile or somatosensory sensations in the arms occurred in 27.3% of EMS stimulation dreams and 11.5% of EMS sham dreams. Balance-related activities were infrequent in both EMS conditions (9.1% in stimulation; 7.7% in sham). For HS, arm movements were reported in 21.4% of dreams in the stimulation condition and 25.0% in the sham condition. Tactile or somatosensory sensations in the arms occurred in 14.3% of HS stimulation dreams and 4.2% of HS sham dreams. No balance-related activities were reported in either HS condition. For GVS, balance-related dream content occurred in 10.0% of dreams in the stimulation condition and 12.0% in the sham condition. Arm movements were reported in 10.0% of GVS stimulation dreams and 20.0% of GVS sham dreams, while no tactile or somatosensory sensations in the arms were reported in either GVS condition. Generalized linear mixed-effects analyses revealed no statistically significant effects (Table 3).

**Table 3:** Simple-effects results from GLMM for stimulation versus sham stimulation.

Outcome	Modality	Test statistic	p-value
**Arm movement**	EMS	χ²(1) = 0.85	.356
	HS	χ²(1) = 0.04	.852
**Tactile sensations (arms)**	EMS	χ²(1) = 0.99	.320
	HS	χ²(1) = 1.54	.214
**Balance-related activity**	GVS	χ²(1) = 0.002	.964

χ² values are from likelihood-ratio tests. No fixed effects reached statistical significance. EMS: Electrical Muscle Stimulation, GVS: Galvanic Vestibular Stimulation, HS: Haptic Stimulation, STIM = stimulation condition, SHAM: sham stimulation condition.

For contextualization purposes, excerpts of dream reports with clear dream incorporation for each condition are presented in Table 4.

**Table 4:** Dream Incorporation Excerpts.

Stimulus	Condition	Scale	Dream Report
EMS	STIM	Movement of the Arms	‘And I had to stick those adhesive strips on my hand with UV-resistant adhesive tape.’
EMS	SHAM	Movement of the Arms	‘...it was just a feeling, and I mostly used my right hand to remove, like to move away this white matter. I was just like constantly trying to move it away.’
HS	STIM	Movement of the Arms	‘Then we were just cleaning it. And now I’ve just been to the fridge and taken out drinks.’
HS	SHAM	Movement of the Arms	‘...and it was like a box of frozen things. And I started packing them...’
GVS	STIM	General Balance-Related Activities	‘I was on a mission, and there we were outside, and we were walking up the mountain, but it was shaky.’
GVS	SHAM	General Balance-Related Activities	‘...and then I fell over. And when I fell over, I fell over into my mother...’
HS	STIM	Tactile or Somatosensory Sensations in the Arms	‘I had my Apple Watch with me, and at first I thought it was vibrating.’
HS	SHAM	Tactile or Somatosensory Sensations in the Arms	‘I had to hold the microphone with two hands. I think holding the microphone definitely was something that had a vibration, like an electronic feeling.’
EMS	STIM	Tactile or Somatosensory Sensations in the Arms	...then suddenly I had to get up, and I had something on my wrist.’
EMS	SHAM	Tactile or Somatosensory Sensations in the Arms	And there was a couple with a young black labrador. And then I was allowed to pet it.’

EMS: Electrical Muscle Stimulation, GVS: Galvanic Vestibular Stimulation, HS: Haptic Stimulation, STIM: stimulation condition, SHAM: sham stimulation condition.

## 4. Discussion

This study tested whether three forms of somatosensory stimulation applied during REM sleep modulate modality-specific dream content under controlled sham conditions. Although bodily movements and sensations were frequently reported across the experimental nights, none of the three stimulation modalities produced a statistically detectable increase in the corresponding dream content categories compared with sham stimulation. These findings should not be interpreted as evidence that external bodily stimuli can never be incorporated into dreams. Rather, they indicate that modality-specific incorporation was not reliably distinguishable from spontaneously occurring bodily dream content under the stimulation parameters and experimental conditions used in the present study. By directly comparing stimulation with modality-matched sham conditions, the study therefore identifies important boundary conditions for experimentally induced dream incorporation. It highlights the need to distinguish genuine stimulation-specific effects from bodily experiences that arise independently within dreams.

### 4.1 Evaluation of stimulus incorporation

#### 4.1.1 Galvanic vestibular stimulation.

Based on prior research on vestibular dreams [19,21,29], GVS was hypothesized to influence balance-related dream content; however, the ‘General Balance-Related Activities’ scale did not confirm this hypothesis.

Observed incorporation rates differed from those reported in an earlier study employing whole-body vestibular stimulation [19]. The present results therefore indicate that, under the stimulation parameters employed here, electrically induced vestibular stimulation was not sufficient to reliably modulate vestibular dream content beyond the level observed during sham stimulation. One possible explanation is that vestibular stimulation during REM sleep must compete with internally generated vestibular experiences, which are common features of dreaming and contribute to sensations of movement, floating, or changes in body orientation [30]. Consequently, subtle externally applied vestibular signals may be insufficient to perturb the internally generated vestibular signals that characterize REM sleep. Moreover, vestibular processing during REM sleep is unlikely to simply mirror vestibular processing during wakefulness, as the internally generated nature of REM consciousness may alter the weighting of incoming vestibular information [31,32]. This interpretation is consistent with the notion that dream experiences are primarily constructed from endogenous neural activity rather than being direct reflections of peripheral sensory input. Interestingly, our recent findings further demonstrated that GVS increased lucid dreaming without increasing vestibular dream incorporation [29], suggesting that vestibular stimulation may preferentially influence higher-order cognitive aspects of dreaming rather than directly modifying vestibular dream content. Several methodological factors should also be considered when interpreting these findings. Pilot testing revealed that the effects of bipolar binaural sinusoidal GVS varied depending on sleeping position and head orientation, making vestibular stimulation during natural sleep inherently difficult to standardize. Furthermore, stimulation intensity and duration were selected to minimize sleep disruption while remaining perceptible; therefore, the present findings apply only to the parameter range investigated. Future studies should systematically explore different stimulation intensities, waveforms, durations, electrode configurations, and head orientations to determine whether stronger or more precisely targeted vestibular stimulation can more effectively influence dream content while preserving stable REM sleep.

#### 4.1.2 Electrical muscle stimulation.

EMS was hypothesized to increase dream content related to arm movement and somatosensory sensations in the arm. However, no statistically significant differences between stimulation and sham conditions were observed for either Movements of the Arms or Tactile or Somatosensory Sensations in the Arms. These findings suggest that the peripheral muscle contractions elicited by EMS, under the stimulation parameters employed in the present study, were insufficient to reliably influence modality-specific dream content beyond the level observed during sham stimulation. One possible interpretation is that externally generated muscle activation does not necessarily translate into a corresponding alteration of the dreamed body. During REM sleep, the experience of movement appears to depend primarily on internally generated sensorimotor representations rather than on peripheral motor activity alone [8,11]. Consequently, externally evoked muscle contractions may be integrated only when they exceed a certain perceptual threshold or coincide with physiological states that increase sensitivity to external input. Whether stronger or differently timed muscle activation would enhance dream incorporation remains an important question for future research. One methodological consideration specific to EMS is that stimulation-induced movement could not be objectively verified during sleep, as blanket coverage and sleeping position may have obscured visible limb movements. Future studies could incorporate objective movement monitoring to clarify the relationship between delivered stimulation, resulting motor output, and dream content.

#### 4.1.3 Haptic stimulation.

HS stimulation did not differ from sham for ‘Movement of the Arms’ or ‘Tactile or Somatosensory Sensations in the Arms’. One possible explanation is that localized tactile stimulation is a familiar sensory experience readily accommodated in ongoing dream generation. The wrist-based haptic stimulation used in the present study represents a relatively localized and familiar type of tactile sensation. A vibrational stimulus on the ankle of participants was previously applied to induce lucid dreaming [18]. However, the absence of sham controls limits direct comparison. The comparable incorporation rates observed during stimulation and sham conditions in the present study therefore suggest that at least under the stimulation parameters employed here, wrist-based vibrotactile stimulation did not reliably produce modality-specific dream incorporation. Future studies should investigate whether alternative stimulation sites, vibration characteristics, or adaptive stimulation paradigms enhance the salience of tactile signals while maintaining stable REM sleep.

### 4.2 Possible physiological mechanisms limiting incorporation

The absence of modality-specific effects across all three stimulation paradigms suggests that externally applied bodily stimulation may be subject to fundamental physiological constraints during REM sleep. Dream experiences are thought to arise primarily from internally generated neural activity, while processing of external sensory input is substantially attenuated during REM sleep [31,32]. In addition, the dreaming brain continuously generates an internal representation of the body that supports vivid experiences of movement, touch, and balance independently of actual sensory input. Within predictive processing accounts of dreaming, internally generated predictions are thought to dominate conscious experience during REM sleep, whereas incoming sensory information is relatively downweighted [32–34]. Consequently, externally delivered bodily stimulation may need to exceed a critical threshold of salience or occur during particularly receptive phases of REM sleep [35] to modify these ongoing internally generated representations rather than simply activating peripheral sensory receptors.

This interpretation may be particularly relevant for vestibular stimulation. Sensations of floating, falling, and altered body orientation are common features of dreaming and may therefore compete with externally induced vestibular input [19,30]. Together, these findings suggest that successful dream incorporation depends not only on the stimulation modality but also on factors such as stimulus salience, REM sleep physiology, and individual differences in sensory responsiveness. Future studies should systematically investigate these factors to define better the conditions under which external bodily stimulation can reliably influence dream content.

### 4.3 Methodological considerations and limitations

Several methodological considerations should be taken into account when interpreting the present findings. First, participants were blinded to the order of stimulation and sham conditions but not to the stimulation modality itself, as all three stimulation devices were attached during each experimental night. Complete double blinding was not feasible because GVS and EMS generated characteristic EEG artifacts, allowing the experimenter to identify stimulation periods. Furthermore, stimulation intensity was calibrated before sleep rather than during REM sleep, although individual arousal thresholds may change across the night. Future studies may benefit from adaptive stimulation algorithms that dynamically adjust stimulation intensity according to physiological state, for example whether stimulation occurs during tonic or phasic REM sleep and the individual’s current arousal threshold [36]. A second consideration concerns the assessment of dream incorporation. The dream report collection after the REM awakenings was relatively simple, consisting of an open question and a direct question targeting the stimulus specifically. More detailed self-report scales may improve sensitivity to subtle incorporation effects [11]. In dream content analysis, a distinction is commonly made between direct and indirect incorporation. Direct incorporation, such as a stimulus being mirrored in the dream (e.g., vibrating finger), is relatively easy to identify [18]. In contrast, indirect incorporation is inherently more ambiguous, as the same stimulus may manifest in diverse and symbolic ways, such as whole-body sensations, environmental events (e.g., earthquakes), or sudden scene changes. For example, petting an animal simultaneously involves arm movement and tactile sensation, meaning that the *Movements of the Arms* and *Tactile or Somatosensory Sensations in the Arms* scales were not entirely independent. Finally, the present analyses did not distinguish between tonic and phasic REM sleep, despite evidence that sensory processing and arousal thresholds differ between these REM substates [35,37]. Finally, the present sample consisted primarily of healthy young adults. Because REM sleep architecture and continuity [38], sensory processing and vestibular function [39] may vary across the lifespan and between healthy and clinical populations, they may not generalize directly to older adults or patient groups. Evaluating dream incorporation across more diverse populations therefore represents an important direction for future research.

### 4.4 Importance of rigorous sham-controlled dream incorporation studies

A principal methodological contribution of the present study is the inclusion of modality-specific sham conditions within a repeated-measures design across three forms of somatosensory stimulation. Although sham controls are the gold standard for establishing causal effects, they remain relatively uncommon in dream incorporation research, with many studies relying solely on stimulation conditions or comparisons with baseline dream reports. Without appropriate sham conditions, it is difficult to determine whether reported incorporations reflect genuine effects of external stimulation or spontaneous fluctuations in dream content. The comparable incorporation rates observed during stimulation and sham conditions across all three modalities highlight the importance of distinguishing stimulation-specific effects from non-specific influences. Rather than suggesting that bodily experiences are absent from dreams, these findings indicate that such content can also emerge spontaneously, independently of experimentally effective stimulation, consistent with previous observations of motor, physiological, and vestibular experiences during dreaming [8,12–15,21]. The sleep laboratory itself has previously been shown to influence dream content, with dreams commonly incorporating experimenters, electrodes, laboratory settings, and experimental procedures [40–42]. In recent novel work, laboratory dreaming has been leveraged to induce lucid dreaming [43]. These observations suggest that contextual influences inherent to the experimental setting and potentially participant expectations may contribute to dream content independently of externally delivered stimulation. Consequently, future studies should routinely incorporate modality-matched sham conditions to distinguish genuine stimulation effects from non-specific experimental influences

### 4.5 Broader implications and methodological outlook

The present findings suggest that successful dream incorporation depends not only on the stimulation modality but also on the complex interaction between external sensory input and internally generated dream processes. Future research should therefore focus on optimizing stimulation timing, intensity, and physiological state, while accounting for individual differences in sensory responsiveness and REM sleep dynamics. Such approaches may help identify the conditions under which external bodily stimulation can reliably influence dream content. One limitation of conventional REM awakening paradigms is that they provide only retrospective access to dream experiences. Lucid dreaming offers a promising complementary approach by enabling participants to evaluate external stimulation while the dream is ongoing consciously. Recent demonstrations of two-way communication during lucid dreaming further suggest that future studies may be able to determine in real time whether external stimuli are consciously perceived, incorporated into dream narratives, or remain unnoticed [44,45]. Such approaches may provide valuable methodological advances for dream engineering and the investigation of sensory processing during sleep.

Beyond testing the primary hypotheses, the present study generated a multimodal dataset comprising repeated REM awakenings following three distinct forms of somatosensory stimulation under carefully controlled experimental conditions. This resource provides a valuable foundation for future hypothesis-driven investigations into sensory processing during REM sleep, dream phenomenology, and the continued methodological development of dream-engineering research.

### 4.6 Conclusion

This study systematically compared three forms of somatosensory stimulation during REM sleep using a within-subject design with modality-specific sham controls, a rigorous sham-controlled experimental test of bodily dream incorporation. Although no evidence was found for reliable modality-specific modulation of dream content, these findings define important boundary conditions under which externally applied bodily stimulation influences dreaming. The comparable incorporation rates observed across stimulation and sham conditions underscore the importance of rigorous control procedures and suggest that contextual and expectancy-related factors contribute substantially to bodily dream experiences. Rather than simply demonstrating the absence of stimulation-specific effects, the present findings establish a methodological benchmark for future research on dream incorporation and highlight the need for increasingly rigorous experimental designs to distinguish genuine stimulation effects from non-specific influences. Future studies should focus on improving methodological precision—including optimized stimulation timing, objective physiological verification, and innovative approaches such as lucid dream communication—to elucidate further how external bodily signals interact with the dreaming brain.
